# A Deep-Learning Approach for Vocal Fold Pose Estimation in Videoendoscopy

**DOI:** 10.1007/s10278-025-01431-8

**Published:** 2025-02-12

**Authors:** Francesca Pia Villani, Maria Chiara Fiorentino, Lorenzo Federici, Cesare Piazza, Emanuele Frontoni, Alberto Paderno, Sara Moccia

**Affiliations:** 1 Department of Information Engineering, Universitá Politecnica delle Marche, Ancona, Italy; 2 Department of Otolaryngology—Head and Neck Surgery, ASST Spedali Civili of Brescia, University of Brescia, Brescia, Italy; 3 Department of Political Sciences, Communication and International Relations, Università degli Studi di Macerata, Macerata, Italy; 4 Department of Innovative Technologies in Medicine and Dentistry, Università degli Studi “G. d’Annunzio”, Chieti - Pescara, Italy

**Keywords:** Vocal folds, Heatmap regression, Pose estimation, Videoendoscopy, Deep learning

## Abstract

Accurate vocal fold (VF) pose estimation is crucial for diagnosing larynx diseases that can eventually lead to VF paralysis. The videoendoscopic examination is used to assess VF motility, usually estimating the change in the anterior glottic angle (AGA). This is a subjective and time-consuming procedure requiring extensive expertise. This research proposes a deep learning framework to estimate VF pose from laryngoscopy frames acquired in the actual clinical practice. The framework performs heatmap regression relying on three anatomically relevant keypoints as a prior for AGA computation, which is estimated from the coordinates of the predicted points. The assessment of the proposed framework is performed using a newly collected dataset of 471 laryngoscopy frames from 124 patients, 28 of whom with cancer. The framework was tested in various configurations and compared with other state-of-the-art approaches (direct keypoints regression and glottal segmentation) for both pose estimation, and AGA evaluation. The proposed framework obtained the lowest root mean square error (RMSE) computed on all the keypoints (5.09, 6.56, and 6.40 pixels, respectively) among all the models tested for VF pose estimation. Also for the AGA evaluation, heatmap regression reached the lowest mean average error (MAE) ($5.87^{\circ }$). Results show that relying on keypoints heatmap regression allows to perform VF pose estimation with a small error, overcoming drawbacks of state-of-the-art algorithms, especially in challenging images such as pathologic subjects, presence of noise, and occlusion.

## Introduction

Vocal folds (VF) are muscular structures located in the larynx, which are responsible for vocalization, breathing, and airway protection. Neurological and inflammatory diseases can lead to impaired VF movements of one or both VF, which may jeopardize key physiological functions of the larynx [1]. Diagnostic assessment of VF paralysis and paresis, glottic stenosis, and other neurologic disorders of the larynx are guided by videoendoscopic imaging, through which clinicians identify irregularities in the adduction and abduction of the folds. One insightful metric for understanding VF movements during breathing and speaking is the change in the angle at the anterior commissure of the membranous VF, the anterior glottic angle (AGA). Traditionally, AGA has been assessed by manually marking laryngoscopy videoframes during standard inhalation and vocalization [2], during cough [3], and during specific lung function tests [4]. This examination, besides being subjective, is time-consuming and requires a skilled professional, and it is characterized by high inter- and intra-rater variability [5, 6]. More recently, advancements in deep learning (DL) are paving the way for more generalizable approaches in several different medical image contests [7–9].

Regarding endoscopy, as a prior for AGA computation, DL researchers are focusing on glottic segmentation, gauging motility through folds movement relative to the glottal midline by using fully convolutional networks (FCN), as in [10] which trains and tests a regression model for segmentation of the glottal region on a dataset of 7892 frames extracted from 58 videos of 20 patients, or a U-Net based approach as in [11], which trains an encoder-decoder model to segment the glottal area on 4500 frames from 7 videos of 7 patients. Other methods rely on region of interest detection, as in [12], which proposes a two-stage method involving region of interest detection followed by an FCN to delineate and segment the glottal opening on a dataset of 806 frames from 194 videos. More recently, to overcome the burden of labeling frames for segmentation tasks, researchers have been focusing on VF pose estimation using anatomical landmarks. The use of landmarks is first explored in [13, 14], both works use an open-source DL toolbox (DeepLabCut [15]). The work in [13] focuses on the offline localization of 13 landmarks on the VF edges from 40 laryngoscopy videos of 40 patients, while [14] extends this study to estimate the AGA using 142 videos from 142 patients. These studies, however, rely on point localization, which can be less robust to occlusions, noise, and variations in recording conditions. These constraints highlight the need for more robust and adaptable approaches to VF pose estimation, such as heatmap regression approaches, which produce a probability distribution instead of specific coordinate values. Thus, to address this gap, we propose the first DL approach for VF pose estimation through keypoints detection based on heatmap regression. This approach can provide better robustness to partial occlusion, a valuable feature in videoendoscopy where VF and other anatomical landmarks may be partially obscured. The choice of using a heatmap regression network, instead of a direct regression network, was driven by previous work from different fields [16, 17], and recent applications in laryngoscopy, including glottal midline detection [18] and laser point localization in 3D laryngoscopy [19]. Moreover, recent advancements outside the medical domain have demonstrated the effectiveness of heatmap regression for keypoint detection, as shown in [20] and [21], further supporting our methodological approach. Additionally, approaches such as [22], which employ non-DL-based methods for keypoint detection in laryngoscopy, indicate that machine learning alternatives can be effective in specific cases of VF analysis. The contributions of this paper can be summarized as follows:

- We propose to use DL-based heatmap regression for VF pose estimation relying on three anatomically relevant keypoints, as a prior to estimate AGA.
- We perform our analysis on a new dataset consisting of 471 laryngoscopy images from 124 patients acquired in the actual clinical practice.

**Fig. 1:**
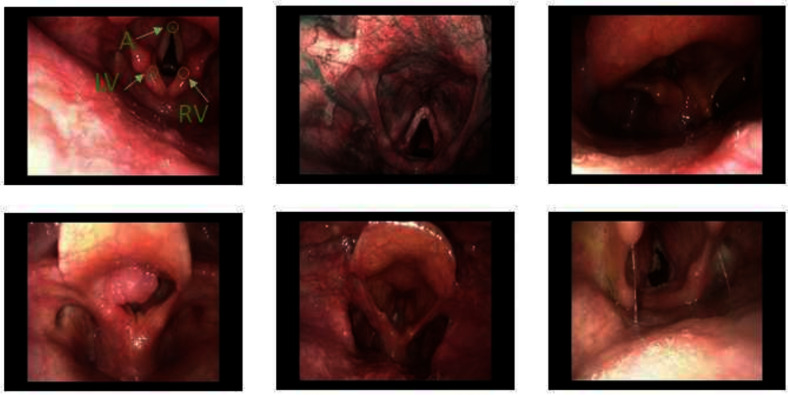
Vocal fold samples. Left (LV) and right (RV) vocal folds, and anterior commissure (A) keypoints are highlighted on the top-left frame. These sample frames show the challenges of the dataset: varying illumination levels, blurring, motion artifacts, the presence of both white-light and narrow-band frames, and frames from pathological subjects (frames in the second row are examples of frames acquired from oncologic patients)

## Materials and Methods

The proposed framework considers three keypoints located at specific sites of the larynx: the posterior angle of the left and right VF (LV and RV, respectively), and the anterior commissure (A), as shown in Fig. 1. The choice of these points is driven by clinical considerations, as the straight lines connecting LV/RV and A delineate the free border of the VF as changes in their position and movement can reveal information about VF angular movements, tension, and adjustments made during voice production.

Our model is inspired by the classical encoder-decoder architecture of U-Net [23]. We employed the MobileNetV2 architecture [24], pre-trained on ImageNet, as the U-Net encoder ($e(\cdot )$), which serves as a feature extractor, and a decoder network ($d(\cdot )$) to recover spatial information and regress heatmaps. We selected MobileNetV2 as ($e(\cdot )$) for its lightweight architecture and computational efficiency, which enable effective feature extraction while remaining suitable for deployment in resource-constrained settings like those in this study. The $e(\cdot )$ is composed of an initial convolutional layer with 32 filters and a stride of 2, which reduces the image size by half, followed by batch normalization and ReLU activation. This initial layer is followed by a series of 17 inverted residual blocks, each consisting of an initial 1 $\times$ 1 convolution followed by a 3 $\times$ 3 depthwise convolution and ending with another 1 $\times$ 1 convolution. At each block, the number of channels increases enabling the incremental learning of more complex features. The number of channels starts from 32 and progressively increases to 576. Similarly, the $d(\cdot )$ is composed of four blocks, each comprising two 2D conv layers followed by batch normalization and ReLU activation. To recover the lost features resulting from the downsampling in the $e(\cdot )$ path, the input of each block is concatenated with the corresponding feature maps from $e(\cdot )$. The last block consists of three 2D conv layers, with the first two being followed by a ReLU activation function, and the last one activated by sigmoid. The proposed architecture is fed by stacking the endoscopic frames and the three corresponding heatmaps of size *W* $\times$ *H* pixels. Each heatmap is represented by a Gaussian distribution centered at the keypoints center.

From the estimated coordinates of the three keypoints, the AGA is computed as follows:

1 $$\begin{aligned} \text {AGA} = \arctan (x_1 \times y_2 - y_1 \times x_2, x_1 \times x_2 + y_1 \times y_2) \end{aligned}$$

where $(x_1, y_1)$ and $(x_2, y_2)$ represent the vector extending from A to LV, and the vector extending from A to RV, respectively.

The dataset used in this study consists of videoendoscopic frames of patients treated at the Unit of Otorhinolaryngology-Head and Neck Surgery, University of Brescia, Italy. Data were acquired using three different Olympus laryngoscopes (models ENF-VH, ENF-VQ, and ENF-V2), following the principles of the Helsinki Declaration, and approval was obtained by the local ethical committee. A total of 471 endoscopic images from 124 patients were collected from a dedicated archive and anonymized. Of these images, 114 were acquired from 28 subjects diagnosed with squamous cell carcinoma. The number of images per patient ranged from 1 to 5 with a median of 3, ensuring a comparable distribution of patient data.

Figure 1 shows some of the challenges in the dataset, including varying illumination levels, presence of both white-light and narrow-band frames, presence of noise, blurring and specular reflection, varying pose of the VF and different fields of view, and frames from pathological subjects. Another challenge in the dataset is related to the AGA variability, which has a median value of $15.12^{\circ }$, with first quartile (Q1)=8.09 and third quartile (Q3)=22.19, and an interquartile range (IQR)=14.10 (minimum AGA value=$1.32^{\circ }$, maximum AGA value=$91.85^{\circ }$).

Frames annotation was performed using Label-studio^1^ by an expert laryngologist with more than ten years of experience. The keypoints were assigned with visibility flags according to the COCO Keypoint detection annotation format: 0 for keypoints not present in the image (which do not occur in our dataset), 1 for keypoints present in the image but not visible (possibly occluded by other anatomical structures), and 2 for clearly visible keypoints.

### Experimental Protocol

All frames were downsampled from their original resolution of 640 $\times$ 480 pixels to 224 $\times$ 224 pixels, and mean intensity was removed from each frame. The ground-truth heatmap was generated by applying a Gaussian function centered at each annotated keypoint, with the intensity of each pixel computed based on its Euclidean distance from the keypoint using the following formula, with $\sigma$ set equal to 20:

2 $$\begin{aligned} f(x,y) = e^{-\frac{(x - x_{\text {center}})^2 + (y - y_{\text {center}})^2}{2 \cdot \sigma ^2}} \end{aligned}$$

The model was trained for a maximum of 200 epochs with early stopping based on validation loss. Training was stopped if the validation loss did not improve for 10 consecutive epochs. Training optimization was performed using the Adam optimizer, with an initial learning rate of 0.001 and a batch size of 8, and a weighted mean squared error (W-MSE) loss $\mathcal {L}$, defined as follows:

3 $$\begin{aligned} \mathcal {L} = \sum _{i=1}^{C} \left( w_i \cdot \frac{1}{N} \sum _{j=1}^{N} (y_{\text {pred}, j, i} - y_{\text {true}, j, i})^2 \right) \end{aligned}$$

where $y_{\text {true}, j, i}$ represents the true value of the $j$-th sample in the $i$-th channel, $y_{\text {pred}, j, i}$ denotes the predicted value of the $j$-th sample in the $i$-th channel, $w_i$ is the weighting factor for the $i$-th channel, $N$ is the total number of samples, and $C$ is the total number of channels. The loss is computed by first calculating the squared differences between the true and predicted values for each channel, multiplying these differences by the respective channel weight, averaging these weighted squared differences for each channel, and then summing these averages across all channels to obtain the total loss. The weights used in this study are $w_{\text {LV}} = 1.2$, $w_{\text {RV}} = 1.2$, and $w_{\text {A}} = 1.0$. The choice of these weights was guided by clinical considerations, emphasizing the importance of LV and RV in defining the free border of the VF. During training, on-the-fly data augmentation was performed to enhance generalization performance. The augmentation techniques included geometrical transformations such as horizontal and vertical flipping, and random rotation in the range of $\pm 30^{\circ }$, and intensity transformations such as random brightness correction in the range [$-$0.1, 0.1], random hue adjustment in the range [$-$0.2, 0.2], and random saturation in the range [0.5, 1.5]. These augmentations were randomly applied at each training iteration. To cope with the small amount of data, and to effectively use all the data available, we performed a five-fold cross-validation. For each fold, images were selected to ensure no patient overlap between the train and test sets. The best model among epochs is selected based on the lowest loss value obtained on the validation set. All the analyses were performed using Tensorflow 2.x on an NVIDIA RTX 2080 TI, with a Xeon e5 CPU and 128 GB RAM.

As ablation study, we investigated the influence of other loss functions on the training outcomes, including the standard mean squared error (MSE) loss and the weighted l1 loss in which the loss is weighted based on the proximity to a certain threshold. Additionally, we compared our framework against direct keypoint coordinate regression, to evaluate the performance differences between them and to demonstrate the advantages of using heatmap regression in terms of robustness in landmark detection. To this goal, we use the same backbone used for heatmap regression (i.e., MobileNetV2 pre-trained on ImageNet) which was followed by a custom regression head made of two separable convolution layers for keypoints coordinate regression. The model was trained using an MSE loss.

A further comparison with literature methods consists of the glottal area segmentation. The model used for this approach is the same as the one used in the proposed heatmap regression method described in the “Materials and Methods” section. In this case, the model is trained for segmentation using the endoscopic frame and the associated mask. The segmentation mask was constructed from the coordinates of the three keypoints: a triangle was formed by the three keypoints LV, A, and RV to define the glottal region mask, assigning to the pixels inside and outside the triangle the values of 1 and 0, respectively. The model was trained using the Dice (*DSC*) loss, defined as L = 1 - *DSC*, where *DSC* is the Dice similarity coefficient, defined as $DSC = \frac{2 \times TP}{2 \times TP + FP + FN}$, where *TP* and *FP* are the true glottal area and background pixels detected as glottal area, respectively, while *FN* refers to glottal pixels that are segmented as background.

For a fair comparison, the ablation study and the comparison with the literature were performed using the same five-fold cross-validation, training setting, and computational hardware. To evaluate the performance of our framework for VF pose estimation and to compare it with the other tested models, we computed the root mean square error (RMSE) [pixels]. In the case of the heatmap regression model, the RMSE was calculated by comparing the coordinates of the ground-truth keypoints and the predicted coordinates, calculated by identifying the positions of the maximum activation value in the predicted heatmaps. In the case of the glottic segmentation model, the coordinates of the keypoints were obtained from the vertices of the smallest enclosing triangle that contains the predicted segmentation masks. The *DSC* was also used as a metric to further assess the segmentation model performance. All metrics were computed at the image level, ensuring an independent evaluation of each prediction.

**Table 1:** Results of the performance metrics computed on the test set of the five folds, and obtained from the proposed heatmaps regression model, the direct regression model, and the glottal segmentation model

Fold	Model	RMSE LV (pixel)	RMSE A (pixel)	RMSE RV (pixel)
*F*0	Heatmap regression	**5.45±13.89**	**8.45±19.38**	**5.60±11.57**
	Direct regression	8.49±6.15	8.66±6.82	7.74±4.70
	Glottal segmentation	6.51±8.46	7.08±6.07	6.55±7.36
*F*1	Heatmap regression	**5.72±13.87**	**5.79±15.13**	**6.66±16.41**
	Direct regression	9.38±7.60	8.44±8.05	10.12±10.43
	Glottal segmentation	8.75±11.56	10.65±9.91	9.03±12.54
*F*2	Heatmap regression	**3.47±3.65**	**5.03±12.46**	**5.17±12.31**
	Direct regression	9.03±7.44	8.96±8.49	7.75±6.49
	Glottal segmentation	6.92±8.95	6.45±6.32	6.68±9.07
*F*3	Heatmap regression	**4.90±13.73**	**4.89±10.68**	**6.89±18.49**
	Direct regression	7.47±6.27	8.66±6.42	7.83±6.24
	Glottal segmentation	7.15±10.64	7.72±7.44	6.89±9.39
*F*4	Heatmap regression	**5.92±12.98**	**8.64±18.81**	**7.70±13.53**
	Direct regression	7.66±6.91	9.74±8.26	8.36±8.03
	Glottal segmentation	6.51±8.98	8.45±10.57	7.04±8.91

Values of the metrics are expressed as mean value (pixels) ± standard deviation

Bold values represent the best results

**Fig. 2:**
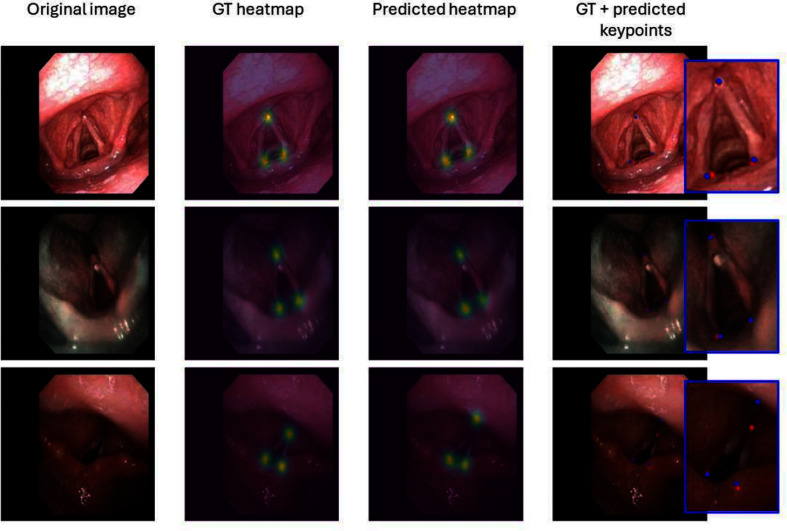
Qualitative results obtained with the proposed framework on three randomly selected test frames. Blue boxes in the last column display close-ups of the (blue) predicted and (red) ground-truth keypoints

**Fig. 3:**
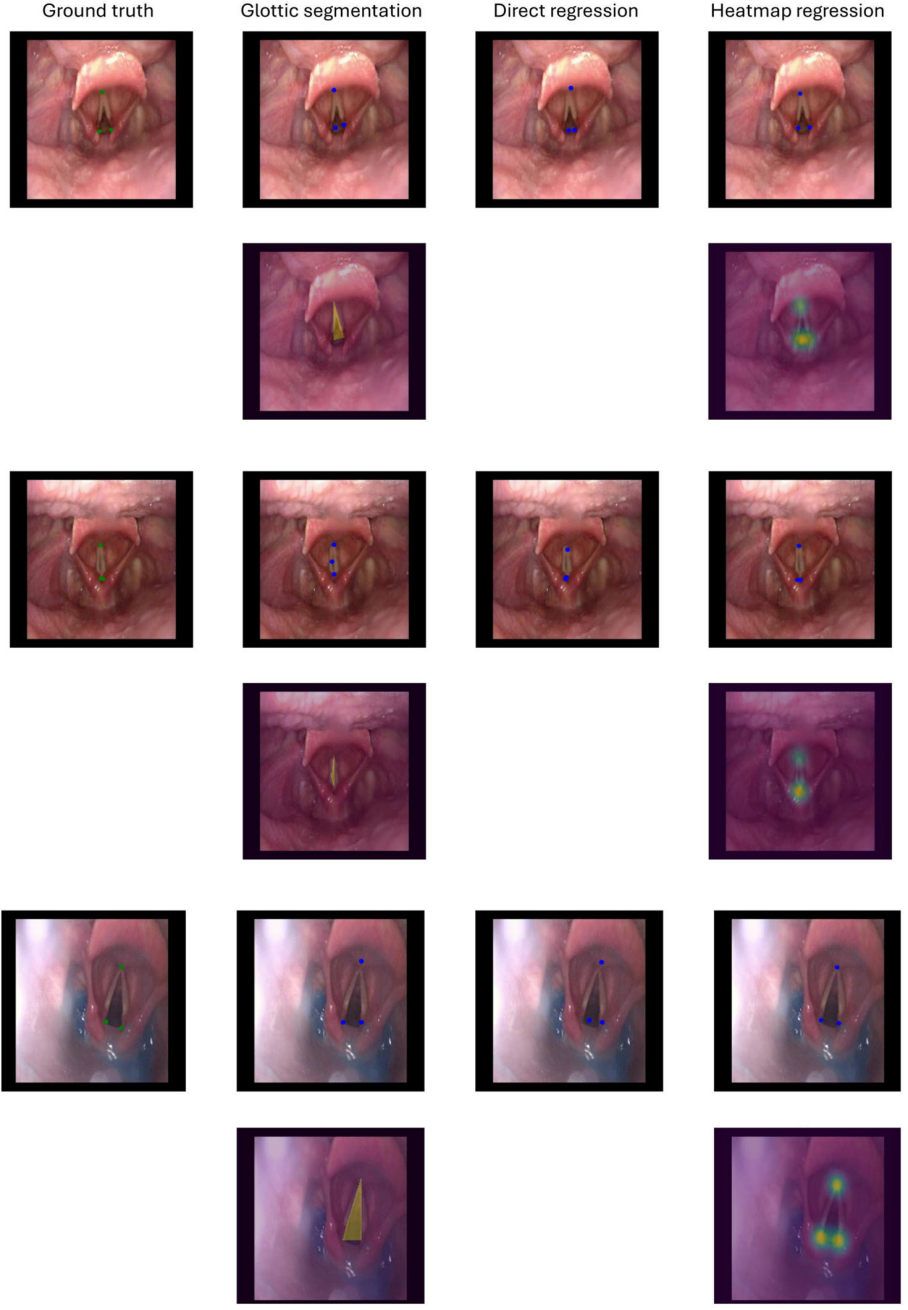
Qualitative results from the comparison of all the tested models on three test samples. The improvements brought by the proposed heatmap regression model are particularly evident in the accurate positioning of the keypoints coordinates, even in more challenging cases, such as reduced glottic opening, or the presence of blur

## Results

### Keypoint Regression

Keypoint regression performance, evaluated in terms of RMSE for all the compared approaches, is reported in Table 1. The proposed heatmap regression model demonstrated consistent and superior performance across the five folds, achieving (mean ± standard deviation) RMSE values of 5.09±11.64, 6.56±15.29, and 6.40±14.46 pixels for LV, A, and RV, respectively. The overall average RMSE across the three keypoints was 6.02 pixels. Qualitative results shown in Fig. 2 highlight the model’s capability to perform well on a variety of challenging images within the dataset, including those with reduced glottic opening or with the presence of blur. To further evaluate the heatmap regression model robustness, its performance was tested separately for oncologic and non-oncologic patients. For non-oncologic patients the mean RMSE resulted to be 4.80±10.13, 5.02±10.04, 5.48±10.62 for LV, A, and RV, respectively, while for oncologic patients, the RMSE resulted to be 5.82±12.28, 11.32±21.96, 8.66±15.38 for LV, A, and RV respectively.

Training the proposed model with different losses resulted in an average performance degradation in terms of RMSE. In particular, training the model with an MSE loss resulted in an average RMSE drop of 0.98 pixels, while the use of the l1 loss shows a higher degradation with an average RMSE delta of 3.73 pixels.

The other two tested models (i.e., direct regression and glottic segmentation) also underperformed compared to the proposed one, as evidenced in Table 1. The direct coordinate regression model performs worse than the proposed one by an average RMSE delta of 2.54 pixels, while the glottic segmentation model shows a higher degradation with an average RMSE delta of 9.95 pixels. Additionally, the *DSC* calculated to evaluate the performance of the glottic segmentation model achieved a mean value across folds of 0.68. In none of the folds, the DSC was higher than 0.70, underscoring the complex nature of endoscopic images. A repeated measures ANOVA revealed a significant main effect of the method on RMSE values ($F(2,8) = 12.10$, $p = 0.0038$, $\eta ^2 = 0.50$), suggesting that the choice of method significantly influences keypoints detection accuracy. Post hoc pairwise comparisons, adjusted using the Holm-Bonferroni correction, demonstrated that the heatmap regression method significantly outperformed the direct method ($p = 0.0227$, Hedges’ $g = 2.76$), with a very large effect size. While the comparison between heatmap regression and glottic segmentation did not achieve statistical significance ($p = 0.0853$), the observed large effect size (Hedges’ $g = 1.20$) suggests a meaningful practical advantage in favor of Heatmap. Similarly, the difference between direct regression and glottic segmentation was not statistically significant ($p = 0.0853$), though a large effect size (Hedges’ $g = 1.08$) indicates glottic segmentation potential superiority over direct regression. Figure 3 shows a comparison of the qualitative results obtained by the tested models, which further supports the effectiveness of the proposed approach in accurately locating the three keypoints compared to the other models even in more challenging cases.

**Table 2:** Mean average error (MAE) and root mean square error (RMSE) of the anterior glottic angle (AGA) computed on the test set of the five folds, for the proposed heatmaps regression model, the direct regression model, and the glottic segmentation model

Fold	Model	MAE (degree)	RMSE (degree)
*F*0	Heatmap regression	**6.39±1.58**	15.01±5.63
	Direct regression	8.74±1.25	13.17±1.56
	Glottic segmentation	8.88±2.56	**11.84±2.41**
*F*1	Heatmap regression	6.54±1.44	14.62±3.42
	Direct regression	13.03±2.35	18.59±2.99
	Glottic segmentation	**5.13±1.00**	**6.87±1.80**
*F*2	Heatmap regression	**4.40±1.65**	9.30±2.19
	Direct regression	9.94±1.56	14.04±2.29
	Glottic segmentation	4.99±0.90	**6.46±0.86**
*F*3	Heatmap regression	**3.52±0.52**	**6.53±1.32**
	Direct regression	9.45±1.73	17.32±3.88
	Glottic segmentation	6.53±0.79	9.44±1.94
*F*4	Heatmap regression	8.48±2.95	22.25±8.10
	Direct regression	10.66±1.52	16.22±3.48
	Glottic segmentation	**5.61±1.55**	**10.12±3.30**

Values are expressed as mean value (degrees) ± standard deviation

Bold values represent the best results

**Fig. 4:**
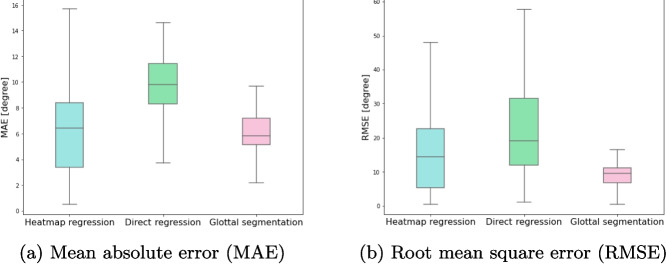
Boxplot of **a** mean absolute error (MAE) and **b** root mean square error (RMSE), calculated on the complete test set, for heatmap regression, direct regression, and glottic segmentation models

### AGA Computation

Table 2 and Fig. 4 summarize the AGA computation performance, evaluated using the regressed keypoints. The heatmap regression model achieved a MAE of 5.87±1.63 and an RMSE of 13.54±4.13. In contrast, the direct coordinate regression model obtained MAE and RMSE values of 10.36±1.68 and 15.87±2.84, respectively. The segmentation model achieved slightly better AGA results, with MAE and RMSE values of 6.23±1.36 and 8.95±2.06, respectively. For the MAE, a repeated measures ANOVA demonstrated a statistically significant main effect of the method on MAE values ($F(2,8) = 9.64$, $p = 0.0074$, $\eta ^2 = 0.632$). Post hoc comparisons revealed that the heatmap regression method significantly outperformed the direct coordinate regression method ($p = 0.0247$, Hedges’ $g = 2.25$), with a very large effect size. While the comparison between heatmap regression and glottic segmentation did not reach statistical significance ($p = 0.7631$), the small effect size ($g = 0.18$) suggests negligible practical differences between these methods under MAE. Also for RMSE, a repeated measures ANOVA revealed no statistically significant differences between methods ($F(2,8) = 4.23$, $p = 0.0557$), though a large effect size ($\eta ^2 = 0.399$) suggests meaningful practical differences. Post hoc pairwise comparisons indicated that the glottic segmentation method significantly outperformed the direct regression method ($p = 0.0437$, Hedges’ $g = 2.77$), while the comparison between the heatmap regression and the glottic segmentation showed a large effect size favoring glottic segmentation ($g = -0.91$), albeit without statistical significance.

As a conclusive analysis, we report the performance of the proposed model on the AGA evaluation for oncologic and non-oncologic patients. It resulted in a mean MAE of 6.85±12.41 and a mean RMSE of 14.46±12.41 in the case of oncologic patients, and a mean MAE of 5.47±11.58 and a mean RMSE of 12.89±11.58.

## Discussion

This research explores the use of a DL method for VF pose estimation in videoendoscopy for AGA estimation. VF pose estimation in endoscopic frames is complicated by the subjectivity of the task leading to variability in image evaluation, and potential inconsistencies in clinical assessments. This study introduces a framework based on keypoints heatmap regression, an effective method to improve model robustness to partial occlusions, ensuring more accurate detection of VF and other anatomical landmarks even when partially obscured. The choice of using a heatmap regression network, instead of a direct regression network, was driven by previous work from different fields [16, 17] as it has been shown that regressing joint positions from an input frame is notably non-linear. By leveraging this approach, the proposed method improved robustness and accuracy compared to other tested models, even in challenging clinical scenarios.

### Keypoint Regression

We achieved robust performance in the estimation of all three keypoints in a newly collected dataset containing patients with pathological conditions. When analyzing the performance relative to pathological subjects, the model shows a higher error in estimating the keypoint at the anterior commissure (11.32±21.96 for oncologic patients, 6.56±15.29 for the general performance). This discrepancy can be attributed to several factors including morphological changes in the VF and surrounding structures caused by oncologic conditions. For instance, tissue irregularities, tumor masses, or post-surgical scarring, as well as occlusion, can obscure the anatomical sites where keypoints are located, causing misplacement. This confirms the difficulty in correctly locating the keypoints of interest in these images, as also shown in the last row of Fig. 2, where the keypoint A is located on the median glosso-epiglottic fold instead of on the anterior commissure.

Evaluating the proposed strategy against the direct keypoint regression model and the segmentation model, the heatmap regression model reached the best performance in terms of RMSE in all five folds. The results of the repeated ANOVA measures conducted on Table 1 also revealed a significant main effect of the method on RMSE values, highlighting the consistent performance of heatmap regression, which achieves significantly lower RMSE values compared to direct regression and demonstrates strong practical advantages over glottic segmentation, particularly in scenarios requiring high precision, such as this clinical case. This result is in line with the literature and can be further appreciated in Fig. 3, where it is shown that the direct regression and the segmentation approaches tend to produce worse keypoints localization, especially in the case in which the folds are adducted and the glottic gap is almost not visible, or in the case of noisy frames, highlighting the superiority of the proposed model in these challenging frames. The heatmap regression method leverages spatial relationships and contextual information to enhance segmentation accuracy, particularly in challenging scenarios involving dark regions. By learning structural priors and predicting probabilistic heatmaps, the approach minimizes reliance on raw pixel intensity and emphasizes the continuity and shape of glottal gaps. This reduces misclassification and improves robustness to lighting and artifact variations.

The lowest performance of the direct regression model may be due to the fact that this approach has reduced spatial context, meaning that directly regressing the point coordinates from the original input image is challenging. On the other hand, the lower performance of the glottic segmentation model compared to the proposed heatmap regression model may be due to the difficulty in localizing the keypoints starting from ground-truth binary masks of the glottis. Moreover, this approach requires a more cumbersome post-processing to extract the coordinates of the keypoints.

**Fig. 5:**
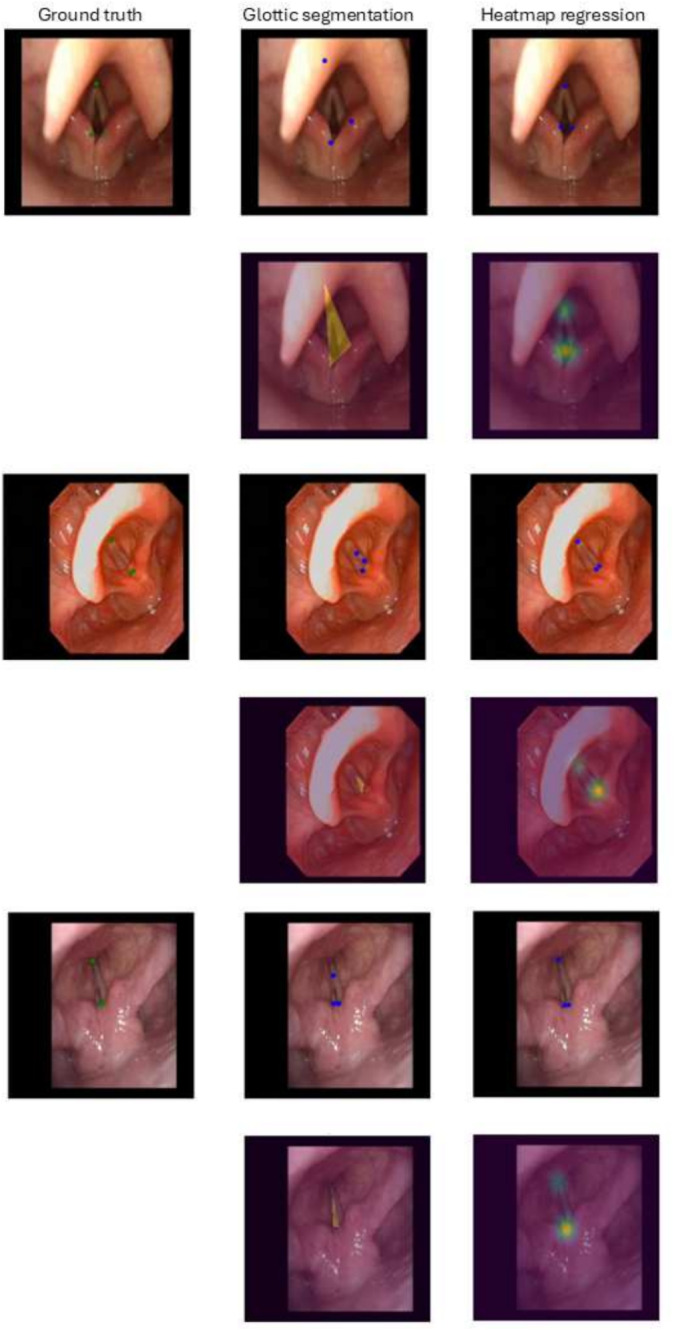
Comparison of keypoint predictions between the heatmap regression and the glottic segmentation approaches on representative endoscopic frames. The heatmap regression approach consistently locates the keypoints closer to the ground truth, even in challenging cases such as the presence of small glottal gaps or keypoints occlusion due to the overlap of other anatomical structures

The proposed model, even when trained with different loss functions (i.e., MSE and l1), consistently achieves better results compared to the other two models it was benchmarked against, underscoring the superiority of our approach. This outcome is particularly important, as precise delineation of anatomical landmarks (VF keypoints in this case) is crucial for accurate diagnosis and treatment planning.

### AGA Computation

Regarding the AGA evaluation, the proposed heatmap regression model shows the lowest average error across the five folds compared to the other tested models, with the only exception for the RMSE which resulted to be lower in the segmentation model. The ANOVA results for AGA and RMSE calculated in Table 2 which also reference Fig. 4 demonstrated a statistically significant main effect of the method on MAE values. Particularly, the heatmap regression method significantly outperformed the direct coordinate regression method, while the comparison between heatmap regression and glottic segmentation did not reach statistical significance. For RMSE, the repeated measures ANOVA revealed no statistically significant differences between methods, even though a large effect size was observed. Post hoc pairwise comparisons indicated that the glottic segmentation method significantly outperformed the direct regression method, while the comparison between the heatmap regression and the glottic segmentation showed a large effect size favoring glottic segmentation, albeit without statistical significance. Despite the lack of statistical significance, it is worth considering other aspects to assess method performance comprehensively. Relative to the latter, the segmentation model demonstrates lower spread and higher performance in terms of MAE and RMSE compared to the heatmap regression approach, but calculating the AGA requires a series of post-processing steps, which introduce additional complexity and potential sources of error. Specifically, once the segmentation mask is obtained, it needs to be approximated to a triangle, from which the vertices are extracted and used to compute the AGA. This process is inherently dependent on the quality and accuracy of the predicted segmentation mask. As highlighted in Fig. 4, the segmentation model shows less variability (lower spread in MAE and RMSE) compared to the heatmap regression approach. However, this robustness comes at the cost of flexibility and feasibility. In cases where the predicted mask represents a very small glottal gap or contains noise, it can be impossible to approximate the region as a triangle. This limitation makes AGA computation infeasible in some cases, due to missing or inaccurate predicted keypoints. By contrast, in the case of the heatmap regression approach, the evaluation of the AGA is performed directly on the predicted keypoints coordinates retrieved by the heatmaps, eliminating the need for such complex post-processing and ensuring consistent results even in challenging scenarios. Figure 5 compares the predicted keypoints of both methods on representative endoscopic frames. As shown, the heatmap regression approach consistently places keypoints closer to the ground truth, even in cases with small glottal gaps or keypoints occlusion due to the presence of other anatomical structures, where the segmentation-based approach struggles to provide reliable predictions. AGA estimation in both oncologic and non-oncologic patients yielded similar results, demonstrating the effectiveness of the proposed model in performing well also in pathological conditions.

While our model exhibits promising results, it also has some limitations. Firstly it was trained and tested on a small dataset; using a larger patient cohort and a higher number of images can improve its ability to correctly estimate VF pose. However, collecting new labeled datasets is not trivial as it requires the involvement of expert clinicians and significant resources in terms of time and cost. The use of generative methods can be beneficial in overcoming this problem by synthetically augmenting the dataset, thus enhancing the model’s training process and improving its overall performance. This approach has already explored in other domains [25], demonstrating its potential to address similar challenges. Furthermore, incorporating public datasets such as BAGLS [26] into a semi-supervised learning framework could help address these limitations by using a larger dataset size to enhance the model’s training process. Even though BAGLS differs from our dataset, as it is not specifically tailored for AGA estimation and lacks keypoint-based annotations on clinically relevant landmarks, its integration could still contribute to improving the generalizability of our pipeline by introducing diverse imaging modalities and broader anatomical variations. A second aspect worth discussing is that we used a MobileNetV2 as the backbone for the heatmap regression model to ensure efficiency but also keep lower computational costs, which are critical factors in clinical applications. While exploring other backbones, such as HRNet, could potentially offer enhanced feature representation capabilities, such backbones generally require significantly higher computational resources and memory, particularly during deployment for inference. This makes them less practical for clinical applications, where real-time or near-real-time processing is often critical. Given the relatively small size of the dataset and our focus on developing an efficient, application-oriented solution, our approach prioritizes speed and efficiency, aligning with the practical constraints of clinical practice while maintaining robust performance. Future development to further extend the current work involves applying a classifier to directly classify the motility of the VF based on their pose estimation, as already presented in a previous work [22]. Another potential direction involves combining segmentation and keypoint-based information in a multi-task framework, enabling the model to simultaneously refine pose estimation and predict motility. This multi-task approach could exploit shared features between tasks, enhancing both segmentation accuracy and classification reliability [18].

## Conclusions

In this paper, we propose a keypoint detection model for VF motility estimation in videoendoscopic images based on heatmap regression. The results achieved on a newly collected dataset suggest that keypoint detection based on heatmap regression can be successfully exploited to estimate VF motility, obtaining better performance compared to direct coordinate regression.

## Footnotes

1 https://labelstud.io/
